# Roles of Indole and Its Derivative in Modulating *E. coli*–*Candida albicans* Biofilm Formation

**DOI:** 10.3390/ijms27104478

**Published:** 2026-05-16

**Authors:** You-Quan Ma, Lan Lin

**Affiliations:** School of Life Science and Technology, Key Laboratory of Developmental Genes and Human Diseases (Ministry of Education, MOE), Southeast University, Nanjing 210096, China; mayouquan@163.com

**Keywords:** *Candida albicans*, biofilm formation, indole, *E. coli*, indole-3-acetic acid (IAA)

## Abstract

*Candida albicans* is the causal agent of invasive candidiasis, which might be lethal in immunocompromised patients. Biofilm formation is considered a key virulence factor of *C. albicans* and is associated with its elevated resistance to antifungals. *C. albicans* and bacteria like *E. coli* are frequently found to form mixed biofilms on biotic or abiotic surfaces, rendering them more refractory to existing antifungals. To investigate how *E. coli* endogenous indole interplaying with exogenous IAA exerts modulatory effects on dual-species biofilm with *C. albicans*, an *E. coli* strain deficient in the indole biosynthetic gene *tnaA* was constructed, and the enzyme TnaA inhibitor was administered to block the indole production in *E. coli* monoculture and/or *E. coli*–*C. albicans* dual culture. Phenotypic assay revealed that indole deficiency attenuated *E. coli* mono-species biofilm by 12% (*tnaA*∆ versus WT *E. coli*), and the lack of indole in the *E. coli* cell-free culture filtrate abolished the ability to promote *C. albicans* biofilms, evidenced by the fact that the treatment with WT *E. coli* culture supernatants exhibited a 1.7-fold promotive effect, while treatment with *tnaA*∆ displayed no significant difference from the broth control towards *C. albicans* biofilms. Furthermore, impaired *E. coli* indole production might disrupt *E. coli*–*C. albicans* biofilm, as examined by confocal laser scanning microscopy (CLSM). Moreover, indole-3-acetic acid (IAA) was found to exhibit more potent biofilm-modulatory activity than indole by CLSM imaging with dual biofilms of WT *E. coli*–*C. albicans*, in contrast to those of *E. coli tnaA*∆–*C. albicans* post-supplemented with exogenous IAA. This study provides evidence for indole as a signaling molecule mediating bacterial–fungal communication during mixed-biofilm formation. Indole and its derivatives, particularly in combination with existing antifungals, have potential in the development of anti-biofilm strategies to eradicate refractory fungal infections.

## 1. Introduction

As a “critical group” among fungal priority pathogens listed by the WHO, *C. albicans* can cause invasive infections (invasive candidiasis) of the blood (candidemia), heart, eyes, and internal organs, with increasingly elevated mortality, especially in immunocompromised patients. Invasive candidiasis has an overall mortality ranging from 20% to 50% despite the availability of active antifungal treatment [1]. Its ability to adhere to surfaces (both biotic and abiotic) and to form biofilms thereon is thought to contribute to the key virulence of *C. albicans* [2]. *C. albicans*, in its biofilm state, has been found to exhibit dramatically increased resistance—by orders of magnitude—to antifungal agents such as azoles and echinocandins while simultaneously evading host immune clearance, thereby rendering *C. albicans* infection extremely difficult to eradicate [3]. Moreover, in such ecological niches as the gastrointestinal tract and implanted medical devices, including catheters and prostheses, *C. albicans* has been found to be in association with bacteria including *E. coli* [4]. Multi-species biofilms are considered to provide more protection against antimicrobial agents and against the actions of the host immune system, and thereby confer advantages to each species over mono-species biofilms [5]. Investigations with cystic fibrosis (CF) patients have reported that species associated with chronic lung infections of CF display elevated resistance in dual-species biofilms in comparison to mono-species biofilms [6]. The recalcitrance to the available therapy of mixed biofilms thus poses a major obstacle in clinical infection control.

Within the intricate architecture of mixed biofilms—often referred to as a “microbial metropolis”—interkingdom signaling networks are critical [7,8]. In complex multiple-species microbial niches, microorganisms can communicate through chemical molecules to sense and respond to the surrounding milieu. Chemical signaling via quorum sensing (QS) has been recognized to modulate the microbial population behaviors, mainly required for surface adhesion as well as niche colonization [8]. Previous studies demonstrated that *Pseudomonas aeruginosa* could adhere to *C. albicans* filaments in response to bacterial QS molecule N-(3-Oxododecanoyl)-L-homoserine lactone, eventually leading to fungal cell death [9,10]. Remarkably, production of this QS molecule might enhance *C. albicans* resistance to the antifungal agent fluconazole by upregulating the efflux pump expression and activating the stress response pathways [11]. Aside from bacterial QS molecules, other metabolites have been revealed to be produced and secreted by *P. aeruginosa*, as exemplified by phenazine, which inhibits *C. albicans* growth at high doses [12] and inhibits hyphal morphogenesis at subinhibitory concentrations [13]. Phenazine production is thought to be at the core of the chemical communication during *C. albicans*–*P. aeruginosa* interaction, thereby leading to the multifaceted effects on the human hosts, for example, CF patients. In the oral cavity, *C. albicans* interacts with the oral opportunistic pathogen *Streptococcus mutans*. This microbe, together with other *Streptococcus* spp., might secrete trans-2-decenoic acid, a small-molecule compound exerting inhibitory action on *C. albicans* hyphal morphogenesis without affecting fungal growth [14]. Moreover, *S*. *mutans* could secrete another small-molecule compound, namely mutanobactin A, which might impede *C. albicans* hyphal morphogenesis [15]. In addition, several bacteria have been reported to secrete soluble factors that might modulate *C. albicans* morphogenesis, such as the opportunistic pathogens *C*. *difficile* and *Burkholderia cenocepacia*, which could produce para-cresol and cis-2-dodecnoic acid, respectively [16,17]. A bi-directional cross-species dialog exists between bacteria and fungi: the bacterial metabolites can profoundly modulate the morphogenesis, virulence expression, as well as the biofilm initiation and development of fungi [18], and vice versa.

Indole, a small-molecule heterocyclic compound produced by *E. coli*, is a widespread and versatile signaling molecule among interspecies microbial communication, for instance, in the human gastrointestinal tract [19,20]. The *E. coli* tryptophanase (TnaA) is responsible for the conversion of tryptophan into indole. Indole has been well-recognized to regulate diverse bacterial physiological processes, including biofilm formation, plasmid stability, antibiotic resistance, and the secretion of virulence factors [21,22,23]. Notably, indole also exhibits eukaryotic activity, acting in a hormone-like manner to alter cell proliferation, host immune responses, and gut barrier function [24,25]. Interestingly, exogenous indole has been found to exert hormetic effects towards multiple species, including bacteria *Aliivibrio fischeri*, *E. coli*, and *Bacillus subtilis*, algae *Microcystis aeruginosa* and *Selenastrum capricornutum*, and human cells like human skin fibroblasts and human cervical cancer cells [26]. In addition, mounting evidence along with our previous studies has demonstrated that indole and its derivative IAA might modulate the hyphal growth in *C. albicans* [27,28], suggesting their potential roles as bacterial messengers in cross-kingdom communication with fungi [26]. In view of that the exerting actions of bacterial endogenously produced indole towards fungal species are less well documented, particularly in the co-culture system of *E. coli* and *C. albicans*, the specific roles of indole or/and IAA within the context of in vivo bacterial–fungal dual-species biofilms are worthy of delving.

Given that current anti-biofilm strategies predominantly target single species [29] and are inadequate against polymicrobial infections, this study is conducted in a model system comprising *E. coli* and *C. albicans*, the two clinically relevant pathogens that are in close association with each other, for mixed-biofilm investigation. It aims to delve into the modulatory effects of bacterial indole on the formation of mono-species as well as dual-species biofilms, and to investigate whether the endogenous indole interplaying with exogenous IAA has impacts on the dual-species biofilms. The findings would pinpoint that indole and its derivative IA]=A have potential as synergistic anti-biofilm agents in combination with existing antifungals to eradicate the biofilm-forming *C. albicans*.

## 2. Results

### 2.1. Construction of an E. coli tnaA Knockout Strain (tnaAΔ) and Complemented Strain

**Figure 1:**
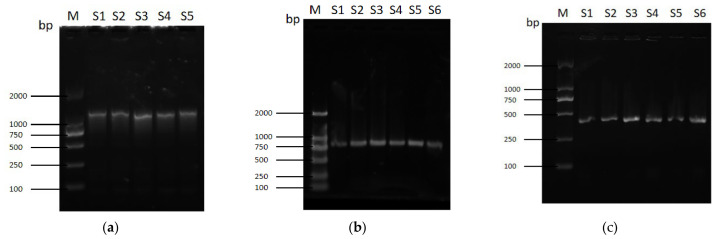
PCR verification of *tnaA* gene knockout (*tnaA*Δ) in *E. coli*. The mutant was constructed via: (**a**) preparation of the targeting DNA fragment, the size of which is 1135 bp as expected, (**b**) replacement of *tnaA* with a selectable marker, showing a DNA fragment of 833 bp as expected, and (**c**) subsequent marker excision, displaying a PCR amplicon of 433 bp as expected. For (**a**–**c**), M indicates DL2000 as a DNA marker; S1–S5 (or S6) represent samples 1–5 (or 6), respectively.

A *tnaA*Δ strain of *E. coli* K-12 BW25113 (*E. coli* WT) was constructed using the λ-Red homologous recombination system. Firstly, PCR amplification of the targeting DNA fragment, consisting of the upstream/downstream homologous arms of *tnaA* and chloramphenicol (Cm) resistance cassette, was conducted (Figure 1a) using the pKD3 as the template with the primers F1/R1 as listed in Supplementary Table S1. Subsequent to its purification, the targeting DNA fragment was introduced into pKD46-harboring *E. coli* WT via electroporation. Transformants were selected for Cm resistance on Cm-containing LB plates. The Cm-resistant transformants were PCR-verified using the respective transformant genomic DNA (gDNA) as the template with the primers F2/R2 as listed in the Table S1, which yielded the amplicon of 833 bp as expected (Figure 1b), indicative of the *tnaA* gene replacement by the Cm resistance (Cm^R^) cassette. The temperature-sensitive helper plasmid pKD46 was eliminated by culturing at a non-permissive temperature, generating genetically stable mutants. Following the excision of Cm^R^ using pCP20, the electrophoresis (Figure 1c) of the PCR amplicon with primers F3/R3 and DNA sequencing analysis (Figure S1) of the resultant clones, designated as *tnaA*∆, confirmed the deletion of the *tnaA* open reading frame (ORF) from the chromosome of *E. coli* WT.

To restore *tnaA* function, a complementation plasmid pSTV28-*tnaA* harboring the full-length *tnaA* with its native promoter was electroporated into the *tnaA*Δ strain, yielding the complemented strain (designated as *tnaA*Δ/pSTV28-*tnaA*). This strain was validated via selection on LB agar plates containing the appropriate antibiotic, followed by plasmid extraction and PCR verification using the primers F4/R4 as shown in Figure S3.

### 2.2. Assessment of Endogenous Indole Production

**Figure 2:**
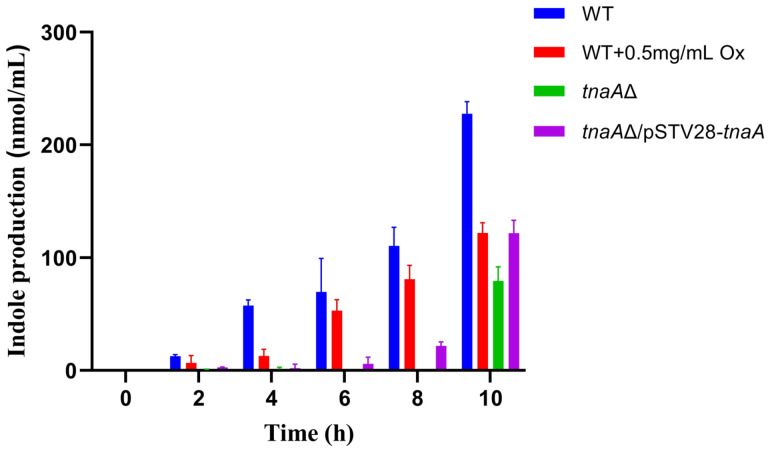
Quantification of indole in bacterial culture supernatants. Indole concentrations were measured in supernatants from wild-type (WT) *E. coli*, *tnaA*Δ, *tnaA*Δ/pSTV28-*tnaA*, and WT cultures supplemented with 0.5 mg/mL oxindole-L-alanine (Ox), respectively. Measurements were taken at 2 h intervals over a 10 h period of growth using the respective time-zero culture as the blank for spectrophotometric analysis (OD_560_).

The ρ-dimethylaminocinnamaldehyde (DMACA) assay revealed that the *E. coli* wild-type (WT) strain exhibited significant accumulation of indole in the culture supernatant after 4 h growth in LB broth, with levels increasing progressively over time, indicating efficient endogenous indole biosynthesis (Figure 2). In contrast, indole levels in the supernatant of *tnaA*Δ culture remained extremely low, consistently below the detection limit during the majority of the monitored period. This indicates that deletion of *tnaA* completely abolishes the endogenous indole-producing ability of the bacterium. Furthermore, indole levels in the *E. coli* complementation strain *tnaA*Δ/pSTV28-*tnaA* were found to increase with the fermentation time going on, though displaying a prolonged lag phase upon inoculation. In addition, WT cultures supplemented with the enzyme TnaA inhibitor oxindolyl-L-alanine (Ox) at 0.5 mg/mL demonstrated substantially low indole yields as compared to the untreated WT counterparts. As previously demonstrated, oxindolyl-L-alanine has been used as a specific inhibitor towards tryptophanase (viz. tryptophan-indole-lyase, TIL) [30,31].

Collectively, these results further pinpoint the essential role of the *tnaA* gene in *E. coli* indole biosynthesis and provide evidence for the modulation of this biosynthetic pathway by exogenous administration of TnaA inhibitors like oxindolyl-L-alanine.

### 2.3. Impacts of Endogenous Indole on E. coli Biofilm Formation

According to the tabulated data, compared to the untreated WT strain, supplementation with varying concentrations of Ox induced a dose-dependent and significant reduction in biofilm formation (Table 1). Specifically, at an Ox concentration of 0.5 mg/mL, biofilm biomass decreased from 0.382 ± 0.035 to 0.331 ± 0.027 (** *p* < 0.01), indicating that Ox could effectively inhibit WT *E. coli* biofilm formation.

Concurrently, the *tnaA* knockout strain (K-12 BW25113 *tnaA*Δ) exhibited significantly lower biofilm formation (0.336 ± 0.054) than the WT strain (*p* < 0.05) without the Ox addition, indicating that the deficiency in indole production due to the loss of the *tnaA* gene might abrogate *E. coli* biofilm-forming capacity. The biofilm-forming level of the complementation strain (*E. coli tnaA*Δ/pSTV28-*tnaA*) was 0.354 ± 0.028, insignificantly different from WT, suggesting the crucial role of the *tnaA* gene in mediating biofilm formation.

Taken together, either the deletion of the indole biosynthetic gene *tnaA* at the genetic level or the supplementation of inhibitor Ox at the TnaA enzyme level significantly inhibited biofilm formation in *E. coli* BW25113. The results from the crystal violet staining assay support that the endogenous indole synthesis pathway in *E. coli* would play an important regulatory role in biofilm formation.

### 2.4. Effects of E. coli Cell-Free Supernatants on C. albicans Biofilm Formation

**Figure 3:**
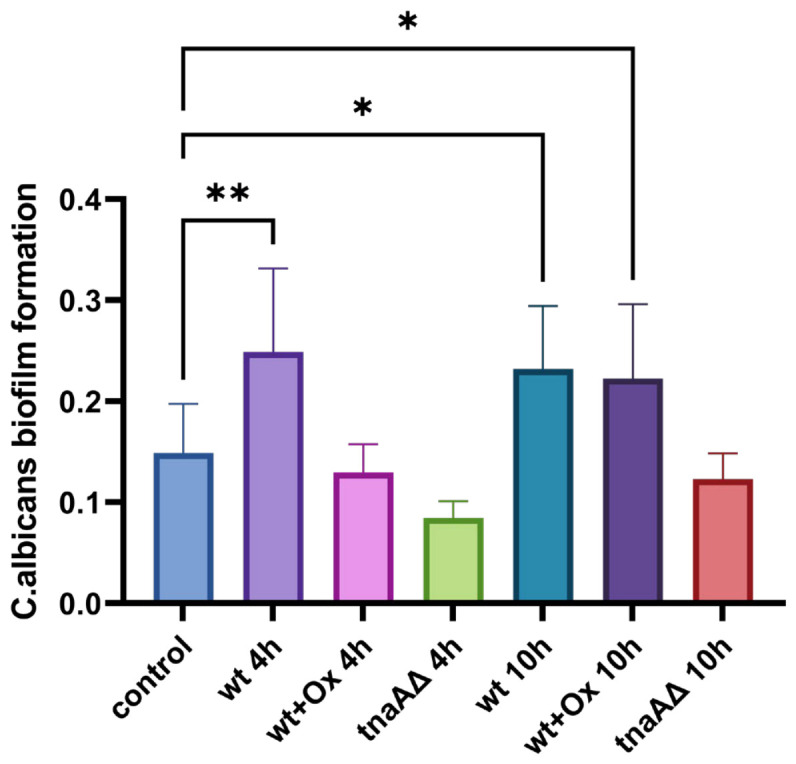
Effects of *E. coli* cell-free supernatants on *C. albicans* biofilm formation. Biofilm formation of *C. albicans* after 48 h treatment with cell-free supernatant from different *E. coli* cultures. Data represent OD_490_ values from three biological replicates for each treatment based on the XTT reduction assay. To normalize for the nutrient residues and pH in the conditioned medium, the absorbency readings of *C. albicans*-inoculated groups were subtracted from those of the un-inoculated control of wt, wt + Ox, and *tnaA*∆, respectively. Asterisks indicate significant differences versus the time-matched wt control (* *p* < 0.05, ** *p* < 0.01, one-way ANOVA with Dunnett’s test).

The methoxynitrosulfophenyl-tetrazolium carboxanilide (XTT) reduction assay revealed that, in comparison to the control group (treated with sterile LB broth), *C. albicans* developed 1.7-fold and 1.6-fold increased biofilms, respectively, while treated with cell-free culture supernatants from WT *E. coli* harvested at different growth phases of 4 h and 10 h. Treatment with the 4 h supernatant from WT *E. coli* grown in the presence of 0.5 mg/mL oxindole-L-alanine (Ox) resulted in *C. albicans* biofilm levels that were not significantly different from the control group. In contrast, treatment with the 10 h supernatant from Ox-supplemented WT *E. coli* cultures led to a significant increase in *C. albicans* biofilm formation. The supernatants of the *tnaA*Δ, regardless of 4 h or 10 h cultures, did not promote *C. albicans* biofilm formation, showing no significant difference from the sterile LB broth control (Figure 3). This indicates that the impairment of endogenous indole production due to the *tnaA* gene knockout in *E. coli* might abolish the ability of its conditioned medium to enhance *C. albicans* biofilm formation.

### 2.5. Transcriptional Analysis of C. albicans Biofilm-Related Genes

**Figure 4:**
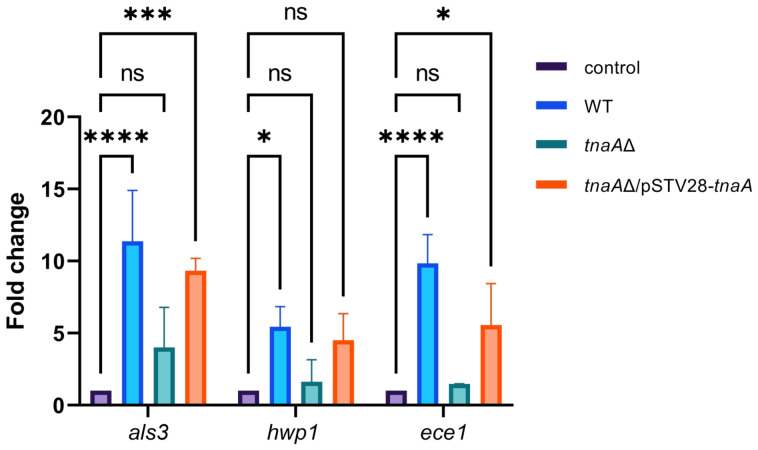
qPCR analysis of *C. albicans* biofilm-associated genes *als3*, *hwp1*, and *ece1* transcripts under the conditions that *C. albicans* cultures were supplemented with *E. coli* cell-free culture supernatants of three genotypes: WT, *tnaA*Δ, and *tnaA*Δ/pSTV28-*tnaA*. RNA extracted from maturation-phase biofilms of *C. albicans* was synthesized into cDNA, followed by the qPCR analysis with the β-actin gene *act1* serving as an internal control for normalization (* *p* < 0.05, *** *p* < 0.001, **** *p* < 0.0001, ns: no significant differences, one-way ANOVA with Dunnett’s test).

In *C. albicans*, the key genes involved in hyphal growth and biofilm formation are *als3* (agglutinin-like sequence 3), *hwp1* (hyphal wall protein 1), and *ece1* (extent of cell elongation 1) [18]. To investigate the effects of the *E. coli*-conditioned medium (also known as cell-free culture supernatants) on the transcripts of the genes associated with *C. albicans* biofilms, RNA extracted from maturation-phase biofilms of *C. albicans* was synthesized into cDNA and subjected to qPCR analysis, where *act1* was used as the housekeeping gene and untreated samples served as the control. As shown in Figure 4, in comparison to the untreated control, treatment with *E. coli* WT-conditioned medium gave rise to the highest increases by 11-, 5-, and 9-fold in transcripts of *C. albicans als3*, *hwp1*, and *ece1*, respectively, among all three treatments herein. Moreover, treatment with *E. coli* complementation strain *tnaA*∆/pSTV28-*tnaA* was found to yield more fold changes in all three gene transcripts than that with the *tnaA*∆ knockout strain, the latter of which were not significantly different from the untreated control (Figure 4).

### 2.6. Effects of Endogenous Indole Interplaying with Exogenous IAA on E. coli–C. albicans Dual-Species Biofilms

Firstly, a *C. albicans* strain was constructed harboring an in-frame expression of eGFP at the C-terminus of adhesion protein Als3 in the chromosome. The homologous recombination fragment for a *C. albicans*-codon-optimized en-hanced green fluorescent protein (yeGFP) integration at the C-terminus of als3 in the *C. albicans* chromosomal locus was shown in Figure S4. Subsequently, the dual-species biofilm-forming experiments were conducted by co-culturing *E. coli* (at an initial cell density of 10^7^ CFU/mL) and eGFP-tagged *C. albicans* (at an initial cell density of ~10^5^ CFU/mL) in a 1:1 (*v*/*v*) mixture of LB and RPMI-1640 liquid medium and incubating for 3 d at 37 °C.

Using one specific label (eGFP) and one unspecific stain, the two consortium members, *C. albicans* and *E. coli*, could be observed and discriminated from each other under confocal microscopy scanning microscopy (CLSM). In accordance with the CLSM exami- as conducted previously with a SYTO^®^ red fluorescent nucleic acid stain, namely Syto 60, in combination with eGFP-expressing strains [32], Syto 63, under this study, did not penetrate eGFP-tagged yeast cells.

**Figure 5:**
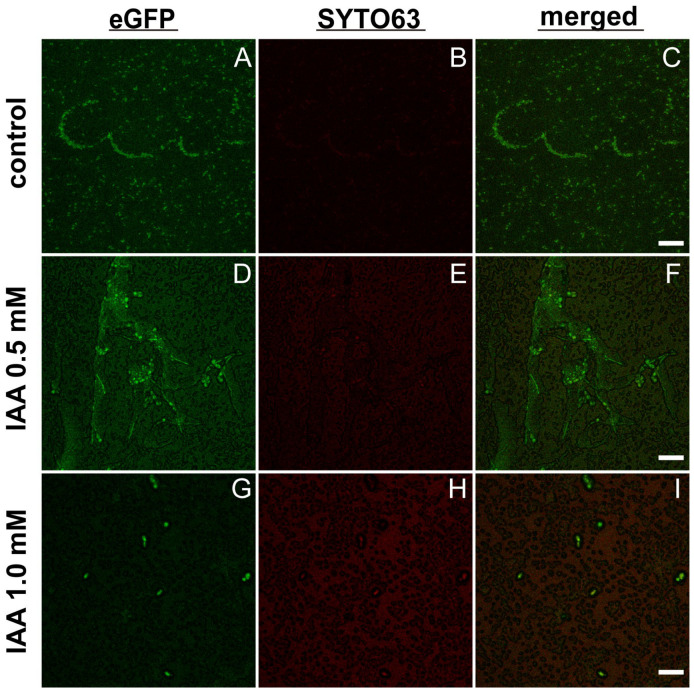
Effects of exogenous IAA on dual-species biofilm initiated with 1:1 (*v*/*v*) mixtures of WT *E. coli* and eGFP-tagged *C. albicans* on the sterile coverslips using the 6-well microplates. Representative confocal images of IAA-untreated control (**A**–**C**), 0.5 mM IAA-treated (**D**–**F**), and 1 mM IAA- treated (**G**–**I**) biofilms, following staining with Syto 63 at 100 nM, taken at the separate channels for eGFP (**A**,**D**,**G**) and Syto 63 (**B**,**E**,**H**), as well as the merged micrographs (**C**,**F**,**I**). Scale bars: 10 µm (**A**–**C**), 20 µm (**D**–**I**).

As shown in Figure 5A–C, in the absence of exogenous IAA, *C. albicans* exhibited hyphal growth to some extent. With the supplementation of IAA at 0.5 mM, *C. albicans* displayed more robust hyphal growth (Figure 5D–F) than the untreated control. With the treatment of a higher IAA (at 1 mM), *C. albicans* was observed to grow in yeast forms predominantly (Figure 5G–H) as compared to that of lower IAA at 0.5 mM (Figure 5D–F) and the untreated control (Figure 5A–C).

**Figure 6:**
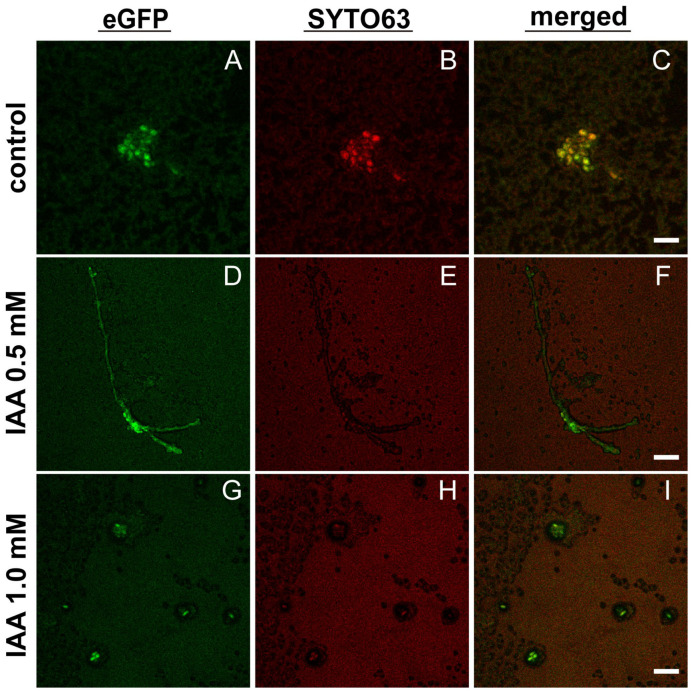
Effects of exogenous IAA on dual-species biofilm initiated with 1:1 (*v*/*v*) mixtures of *E. coli tnaA*∆ and eGFP-tagged *C. albicans*. Representative confocal images of IAA-untreated control (**A**–**C**), 0.5 mM IAA-treated (**D**–**F**), and 1 mM IAA-treated (**G**–**I**) biofilms, following staining with Syto 63 at 100 nM, taken at the separate channels for eGFP (**A**,**D**,**G**) and Syto 63 (**B**,**E**,**H**), as well as the merged micrographs (**C**,**F**,**I**). Scale bars: 10 µm (**A**–**C**), 20 µm (**D**–**I**).

As shown in Figure 6, the dual-species biofilms were examined of *C. albicans* co-cultured with the *E. coli tnaA*∆ strain lacking the indole production. In the absence of exogenous IAA (Figure 6A–C), *C. albicans* displayed mostly in yeast forms, probably owing to the deficiency of indole production by *E. coli tnaA*∆, as compared with those of *C. albicans* co-cultured with WT *E. coli* (Figure 5A–C), which showed some degree of hyphal growth. With the addition of IAA at 0.5 mM, *C. albicans* was observed to initiate hyphal growth (Figure 6D–F) as compared to that of the untreated control (Figure 6A–C), inferring that IAA might possess more potent biofilm-modulatory effects than indole. However, with the supplementation of a higher IAA level at 1 mM, *C. albicans* was found to grow in the yeast forms (Figure 6G–I), which suggests that high IAA might inhibit the yeast–hyphae morphological transition, while low IAA might display stimulatory effects on morphlogical transition from yeast to hyphae in *C. albicans*.

## 3. Discussion

This study investigated the role of the bacterial metabolite indole in regulating biofilm formation, both in mono-species *E. coli* cultures and in dual-species consortia with *C. albicans*. Our findings consolidate and extend the understanding of indole as a key signaling molecule of interkingdom interaction, revealing its complex and context-dependent impacts on microbial community dynamics and virulence.

Firstly, the central role of endogenous indole was validated in *E. coli* biofilm formation. Both genetic ablation of the *tnaA* gene and biochemical inhibition of the TnaA enzyme with oxindole-L-alanine (Ox) significantly impaired biofilm formation in *E. coli*. The restoration of biofilm formation in the genetically complemented strain (*tnaA*∆/pSTV28-*tnaA*) substantiates that the observed phenotype is attributed to the loss of indole production rather than secondary effects of the mutation. This aligns with the previous studies indicating that indole could modulate various bacterial physiological processes, including biofilm architecture and stability [21,22].

Intriguingly, our data revealed a subtle role for indole in the *E. coli*–*C. albicans* cross-kingdom communication. The cell-free supernatant from wild-type *E. coli* could promote *C. albicans* biofilm formation, whereas the supernatant from the *E. coli tnaA*∆ might abolish this stimulatory capacity. This demonstrates that indole, or an indole-regulated factor (receptor, effector, etc.), is necessary for *E. coli* to exert biofilm-promotive actions on *C. albicans*. In addition, this biofilm-promoting effect was dependent on the bacterial growth phase, suggesting a temporal pattern of *E. coli* indole biosynthesis. This is reminiscent of the notion, proposed by Martino et al. [21], that the increased cell density of *E. coli*, along with nutrient depletion, might trigger and activate the key indole biosynthesis gene *tnaA*.

Furthermore, our CLSM imaging studies indicated that an indole derivative such as IAA could exhibit dual effects on *C. albicans*–*E. coli* mixed biofilms—low-dose IAA stimulates the *C. albicans* yeast–hyphal transition (Figure 5D–F *versus* Figure 5A–C) and high-dose IAA inhibits the hyphal growth and reverts to yeast forms (Figure 5 G–I *versus* Figure 5 A–C). This tendency was also manifested in the biofilm studies of *C. albicans* co-cultured with the *E. coli tnaA*∆ mutant by CLSM (Figure 6D–F *versus* Figure 6A–C; Figure 6G–I *versus* Figure 6A–C). In view of that, *C. albicans* yeast–hyphal transition is the prerequisite of its biofilm formation, the dual modulatory effects of IAA on *C. albicans* biofilm formation, as visualized by CLSM, are reminiscent of the previous report indicating that IAA at 200 µg/mL (viz. 1.14 mM) could inhibit *C. albicans* biofilm formation, while IAA at 100 µg/mL (viz. 0.57 mM) exerted promotive effects [28]. Hormesis is widely recognized as a dose-dependent response, featured by promotion in the low-dose range and inhibition in the high-dose range of a given chemical molecule [26]. As proposed by another study regarding the effects of indole towards seven model organisms (three bacteria—*A. fischeri*, *E. coli*, *B. subtilis*, two algae and two human cell lines), the mechanistic elucidation of time-dependent hormetic effects on the bioluminescence of *A. fischeri* revealed that the indole ring might be the central structure responsible for making indole act on the quorum sensing (QS) to induce hormetic phenomenon [26]. More future work would need to be conducted to delve into the molecular mechanism of the hormetic effects of the indole derivative IAA.

Remarkably, exogenous high-dose IAA (1 mM) was found to exert potent anti-biofilm effects, interplaying with endogenous indole produced by *E. coli*, which opens a promising therapeutic avenue. This suggests that the morphological transition from yeast to hyphae in *C. albicans* and the ensuing biofilm formation with *E. coli* is not only dependent upon the indole signaling pathway but also governed by an interweaving network that both indole and IAA participate in. Indole and its derivative IAA, along with synthetic analogs or inhibitors, could modulate the interkingdom communication during mixed-species biofilm formation, thereby offering a novel anti-biofilm strategy [33]. Accumulating evidence has uncovered chemically modified indole derivatives/analogs that might be comparable to or even outperform the existing antifungal agents [34,35,36]. Ma et al. (2022) designed and prepared a series of novel indole and indoline derivatives, among which four compounds exhibited good antifungal effects towards azole-resistant *C. albicans* [36]. Against azole-resistant *Candida* spp., including *C. albicans* and *C. auris*, Jeong et al. (2025) designed and screened 50 multi-halogenated indole derivatives, among which 4,6-dibromoindole and 5-bromo-4-chloroindole exhibited the strongest antifungal and anti-biofilm effects, with minimum inhibitory concentration (MIC) values of 10–50 µg/mL, outperforming ketoconazole and comparable to miconazole [34]. Indole derivatives such as 7-benzyloxyindole could be used to control fungal virulence, as proposed by Manoharan et al. (2018) [35].

Finally, the reciprocal effect of *C. albicans* on *E. coli* physiology within the dual-species mixed biofilm, hinted at in the Results section (Section 2), needs further investigation, thereby facilitating a full understanding of the bidirectional nature of this bacterial–fungal interaction.

In conclusion, this work establishes indole as a crucial bacterial signal that orchestrates both intraspecies (*E. coli*) and interspecies (*E. coli*–*C. albicans*) biofilm development. Its role is multifaceted, regulating bacterial biofilm formation while simultaneously modulating fungal virulence (as indicated by morphologic transition and biofilm formation) in a complex manner, interplaying with exogenously supplemented IAA. These insights underscore the potential of targeting microbial communication networks rather than just viability as a strategy to combat recurrent polymicrobial infections associated with biofilms. Future work should focus on elucidating the fungal sensory machinery for indole and evaluating the efficacy of indole-pathway inhibitors in combination with conventional antifungals in vivo.

## 4. Materials and Methods

### 4.1. Strains, Culture Conditions, and Reagents

The model microorganisms used in this study were as follows: *E. coli* K-12 BW25113 wild-type and its derivative strains, routinely cultured in LB liquid medium at 37 °C; and the standard wild-type (WT) *C. albicans* strain SC5314, cultured in YPD broth at 30 °C [37]. For *C. albicans* biofilm formation assays, the growth and induction medium was switched to RPMI-1640. All chemical reagents used in the experiments, including the TnaA-specific inhibitor oxindolyl-L-alanine (Hanhong Sci. Co., Shanghai, China), indole (Macklin, Shanghai, China), and its derivative IAA (Aladdin Sci., Shanghai, China), were of analytical grade purity.

### 4.2. Gene Disruption of tnaA and Gene Complementation by Plasmid Rescue in E. coli

The *E. coli tnaA* knockout strain (*tnaA*Δ) was constructed in the K-12 BW25113 wild-type background using the λ-Red homologous recombination system [38]. This system utilizes the recombinase expressed from the temperature-sensitive plasmid pKD46, which carries arabinose-inducible *red* recombinase genes and is maintained at 30 °C. Briefly, a linear targeting DNA fragment—flanked by sequences homologous to the regions upstream and downstream of the *tnaA* gene and containing a chloramphenicol resistance marker (Cat)—was electroporated into *E. coli*-competent cells harboring pKD46. Following arabinose induction, λ-Red recombinase-mediated homologous recombination resulted in the replacement of the chromosomal *tnaA* gene with the resistance marker Cat. The temperature-sensitive pKD46 plasmid was subsequently cured by culturing at 42 °C, and putative knockout clones were selected on LB agar plates containing chloramphenicol. The resistance cassette was then excised using the pCP20 plasmid. Finally, the genotype of the mutant was confirmed by colony PCR and DNA sequencing, verifying the successful and precise deletion of the *tnaA* gene. The *tnaA* knockout strain was complemented by transformation with the recombinant vector pSTV28 carrying *tnaA* (viz. pSTV28-*tnaA*).

### 4.3. Quantification of Indole Levels

The indole concentration in bacterial culture supernatants was quantified using a colorimetric assay with p-dimethylaminocinnamaldehyde (DMACA) as the chromogenic reagent [39]. Briefly, a standard curve was generated using a series of indole standard solutions of known concentrations. The target bacterial strains were inoculated into fresh medium at an initial OD_600_ of 0.01. Samples were collected every 2 h during cultivation. Following centrifugation and 0.22 µm filtration, the cell-free supernatant was reacted with the DMACA reagent, and the absorbance of the reaction product was measured at 560 nm. The indole concentration in the samples was subsequently calculated based on the standard curve (Figure S2).

### 4.4. Biofilm Biomass Quantification by Crystal Violet Staining

Biofilm biomass was quantified using a 96-well microplate crystal violet staining assay [40]. Briefly, bacterial suspensions in LB medium were inoculated into flat-bottomed sterile 96-well polystyrene microplates and incubated statically at 37 °C for 24 h to allow biofilm formation. After incubation, the culture medium was aspirated, and non-adherent planktonic cells were removed by gently washing the wells three times with phosphate-buffered saline (PBS). Crystal violet solution (0.1%) was then added to cover the well bottoms and incubated for 30 min at room temperature. The stain was discarded, and unbound dye was removed by rinsing with deionized water. After the plates were air-dried, 33% acetic acid solution was added to solubilize the crystal violet bound to the biofilm. Finally, the solubilized dye was transferred to a new microplate, and the absorbance at a specific wavelength (550 nm) was measured using an iMark^TM^ microplate reader (BioRad, Hercules, CA, USA). This absorbance value serves as an indirect measure of the total biofilm biomass. All assays were conducted in triplicate for each treatment.

### 4.5. Metabolic Activity Assay–XTT Reduction Assay

The metabolic activity of cells within the biofilm was assessed using the XTT (2,3-Bis-(2-Methoxy-4-nitro-5-sulfophenyl)-2H-tetrazolium-5-carboxanilide) reduction assay, following a previously described protocol with slight modifications [41]. An XTT-menadione solution was freshly prepared by dissolving XTT in pre-warmed Ringer’s solution to a final concentration of 0.5 mg/mL, followed by the addition of menadione (10 µM) as an electron-coupling agent. After biofilm formation and washing steps (as described in Section 4.4), 100 µL of the XTT-menadione solution was added to each well. The 96-well microplate was then incubated in the dark at 37 °C for 2 h. Following incubation, 80 µL of the supernatant from each well was carefully transferred to a new 96-well microplate. The absorbance of the formed formazan product, which correlated with cellular dehydrogenase activity, was measured at 490 nm using an iMark^TM^ microplate reader (BioRad, Hercules, CA, USA). All assays were performed with a minimum of three biological replicates.

### 4.6. Effect of E. coli Cell-Free Supernatant on C. albicans Biofilm Formation

To investigate the impact of bacterial metabolites on fungal biofilm, culture supernatants from *E. coli* at different growth phases (logarithmic and stationary phases) were collected. The cultures were centrifuged and sterilized by membrane filtration to prepare sterile cell-free supernatants, which were used immediately. *C. albicans* was inoculated at an initial OD_600_ of 0.1, allowed to pre-adhere for 1.5–2 h, and then washed with PBS to remove non-adherent cells. Subsequently, an equal volume of RPMI-1640 medium was mixed with the *E. coli* cell-free supernatant, and the mixture was added to the wells to induce biofilm maturation over 48 h. Biofilm quantification was performed as described above.

### 4.7. Quantitative Real-Time Polymerase Chain Reaction (qPCR) of C. albicans Biofilm-Associated Genes

*C. albicans* was inoculated onto sterile polystyrene flat-bottomed six-welled microplates with an initial OD_600_ of 0.1, allowed to pre-adhere for 2 h, and then washed with PBS to remove non-adherent cells. Subsequently, an equal volume of RPMI-1640 medium was mixed with the *E. coli*-conditioned medium (harvested at the late logarithmic phase of *E. coli* culture, followed by centrifugation, with the supernatants filtered through 0.22 µm filters), and the mixture was added to the individual well of the microplate to induce biofilm maturation over 48 h.

Total RNA from *C. albicans* maturation-phase biofilms was extracted and synthesized into cDNA using PrimeScript™ RT Master Mix (Perfect Real Time, Takara Bio, Kyoto, Japan) based on the manufacturer’s instructions. qPCR was carried out using TB Green^®^ Premix Ex Taq™ II (Takara Bio, Kyoto, Japan) with the primers of *C. albicans* biofilm-associated gene *als3*, *hwp1*, and *ece1* (listed in Table S2) on the Applied Biosystems™ QuantStudio™ 5 real-time PCR instrument (Thermo Fisher Scientific, Waltham, MA, USA).

### 4.8. Biofilm Examination by Confocal Laser Scanning Microscopy (CLSM)

For biofilm visualization, imaging analysis was performed using CLSM [42]. First, a *C. albicans* strain expressing a fused eGFP protein was constructed. The fusion protein consisted of a *C. albicans*-codon-optimized enhanced green fluorescent protein (yeGFP) tagged to the C-terminus of adhesion protein Als3. Dual-species biofilms of *E. coli* and *C. albicans* were prepared on sterile glass coverslips and placed in 6-well microplates, as previously described with modification [43,44,45]. Briefly, overnight cultures of *E. coli* (~10^7^ CFU/mL) and yeGFP-expressing *C. albicans* (~10^5^ CFU/mL) were inoculated in RPMI 1640 medium (final volume 2 mL) in the presence or the absence of IAA (0, 0.5, 1 mM), followed by static incubation at 37 °C for 72 h. To visualize *E. coli* within the biofilm, the coverslips were washed with 0.9% NaCl to remove planktonic cells and were subsequently stained with SYTO 63 dye (Invitrogen, Carlsbad, CA, USA) at the working concentration of 100 nM for 10 min at room temperature, followed by washing with 0.9% NaCl to remove unbound dye. The biofilms were imaged using confocal laser scanning microscopy, LSM 700 (Zeiss, Oberkochen, Germany). The ZEISS ZEN 3.13 software was used to process the micrographs. Three independent cultures were carried out for each experimental treatment, and at least 10 random positions were assayed.

### 4.9. Statistics

All experiments were performed with at least three independent replicates. The results are presented as the mean ± standard deviation. Statistical analysis was performed using GraphPad Prism software 9.5.0 (GraphPad, Boston, MA, USA). Comparisons between groups were conducted using a Student’s *t*-test or one-way analysis of variance (ANOVA). A *p*-value of less than 0.05 was considered statistically significant.

## 5. Conclusions

This study establishes the bacterial tryptophan metabolite indole as a critical signaling molecule that orchestrates both intraspecies and interkingdom biofilm dynamics between *Escherichia coli* and *Candida albicans*. Endogenous indole is essential for *E. coli* mono-species biofilm formation, and it, through diffusion in/out of the bacterial plasma membrane, also mediates bacterial–fungal communication, as the indole-deficient *E. coli* supernatants were found to lose the capacity to stimulate *C. albicans* biofilm formation. While our in vitro data suggest that targeting the indole/IAA signaling pathways could be a promising anti-biofilm strategy, its clinical translation approach encounters significant hurdles. Future work must validate these findings in relevant animal models to confirm efficacy within a host environment. A key limitation is the narrow therapeutic window and potential host cytotoxicity of these metabolites, as their effects are concentration-dependent. Therefore, pharmacokinetic/pharmacodynamic studies and the development of more selective indole analogs or TnaA inhibitors with improved safety profiles are essential, as demonstrated by the design of indole derivatives with significantly enhanced potency and specificity. Furthermore, effective delivery strategies (e.g., via nano-carriers or liposomal-based vesicles) are needed to ensure targeted delivery and sufficient concentration at the infection site [46]. The promising in vivo efficacy of indole derivatives against multi-species biofilms, including those involving *C. albicans*, underscores the potential of this approach. Addressing these translational considerations—through integrated efforts via in vivo validation, medicinal chemistry, and advanced drug delivery—will be crucial for developing this strategy into a feasible therapeutic approach. Interestingly, while enhancing overall *C. albicans* biofilm formation, indole’s promotive effects could be fortified by a low dose of exogenous IAA (Figure 5D–F and Figure 6D–F) but masked by high-dose IAA supplementation, the latter of which could revert the *C. albicans* hyphal to yeast growth (Figure 5G–I and Figure 6G–I), thereby inhibiting *C. albicans* biofilm. These findings highlight that targeting the indole as well as IAA signaling pathways could be a novel anti-virulence strategy, particularly in combination with existing antifungals, against refractory biofilm-associated *C. albicans* infections.

## Figures and Tables

**Table 1:** Effects of oxindolyl-L-alanine (Ox) and *tnaA* on *E. coli* biofilm formation.

*E. coli* Strain	Growth Medium Supplementation	Biofilm Formation
K-12 BW25113	—	0.382 ± 0.035
	Ox (0.125 mg/mL)	0.352 ± 0.014 *
	Ox (0.25 mg/mL)	0.350 ± 0.031 *
	Ox (0.5 mg/mL)	0.331 ± 0.027 **
K-12 BW25113 *tnaA* Δ	—	0.336 ± 0.054 *
K-12 BW25113 *tnaA*Δ/pSTV28-*tnaA*	—	0.354 ± 0.028

All experiments were performed in Luria–Bertani (LB) medium supplemented with oxindolyl-L-alanine and no supplementation. Crystal violet staining quantification (OD_550_). * *p* < 0.05 compared to WT, ** *p* < 0.01.

## Data Availability

The original contributions presented in this study are included in the article/Supplementary Materials. Further inquiries can be directed to the corresponding author.

## Supplementary Materials

The following supporting information can be downloaded at: https://www.mdpi.com/article/10.3390/ijms27104478/s1.

## Abbreviations

CLSM	confocal laser scanning microscopy
LB	Luria–Bertani broth
PBS	phosphate-buffered saline
RPMI	Rosewell Park Memorial Institute
XTT	methoxynitrosulfophenyl-tetrazolium carboxanilide
YPD	Yeast extract peptone dextrose

## References

[1] World Health Organization WHO Fungal Priority Pathogens List to Guide Research, Development and Public Health Action. https://www.who.int/publications/i/item/9789240060241.

[2] Lohse M.B., Gulati M., Johnson A.D., Nobile C.J. (2018). Development and regulation of single- and multi-species *Candida albicans* biofilms. Nat. Rev. Microbiol..

[3] Wall G., Montelongo-Jauregui D., Vidal Bonifacio B., Lopez-Ribot J.L., Uppuluri P. (2019). *Candida albicans* biofilm growth and dispersal: Contributions to pathogenesis. Curr. Opin. Microbiol..

[4] Bachtiar E.W., Bachtiar B.M. (2020). Effect of cell-free spent media prepared from *Aggregatibacter actinomycetemcomitans* on the growth of *Candida albicans* and *Streptococcus mutans* in co-species biofilms. Eur. J. Oral. Sci..

[5] Burmølle M., Ren D., Bjarnsholt T., Sørensen S.J. (2014). Interactions in multispecies biofilms: Do they actually matter?. Trends Microbiol..

[6] Lopes S.P., Ceri H., Azevedo N.F., Pereira M.O. (2012). Antibiotic resistance of mixed biofilms in cystic fibrosis: Impact of emerging microorganisms on treatment of infection. Int. J. Antimicrob. Agents.

[7] Marcomini E.K., Negri M. (2023). Fungal quorum-sensing molecules and antiseptics: A promising strategy for biofilm modulation?. Drug Discov. Today.

[8] Oliveira R.A., Cabral V., Torcato I., Xavier K.B. (2023). Deciphering the quorum-sensing lexicon of the gut microbiota. Cell Host Microbe.

[9] Hogan D.A., Kolter R. (2002). Pseudomonas-Candida interactions: An ecological role for virulence factors. Science.

[10] Hogan D.A., Vik Å., Kolter R.A. (2004). *Pseudomonas aeruginosa* quorum-sensing molecule influences *Candida albicans* morphology. Mol. Microbiol..

[11] Bandara H.M.H.N., Wood D.L.A., Vanwonterghem I., Hugenholtz P., Cheung B.P.K., Samaranayake L.P. (2020). Fluconazole resistance in *Candida albicans* is induced by *Pseudomonas aeruginosa* quorum sensing. Sci. Rep..

[12] Gibson J., Sood A., Hogan D.A. (2009). *Pseudomonas aeruginosa Candida albicans* interactions: Localization and fungal toxicity of a phenazine derivative. Appl. Environ. Microbiol..

[13] Morales D.K., Grahl N., Okegbe C., Dietrich L.E., Jacobs N.J., Hogan D.A. (2013). Control of *Candida albicans* metabolism and biofilm formation by *Pseudomonas aeruginosa* phenazines. mBio.

[14] Vílchez R., Lemme A., Ballhausen B., Thiel V., Schulz S., Jansen R., Sztajer H., Wagner-Döbler I. (2010). *Streptococcus mutans* inhibits *Candida albicans* hyphal formation by the fatty acid signaling molecule trans-2-decenoic acid (SDSF). ChemBioChem.

[15] Joyner P.M., Liu J., Zhang Z., Merritt J., Qi F., Cichewicz R.H. (2010). Mutanobactin A from the human oral pathogen *Streptococcus mutans* is a cross-kingdom regulator of the yeast-mycelium transition. Org. Biomol. Chem..

[16] van Leeuwen P.T., van der Peet J.M., Bikker F.J., Hoogenkamp M.A., Oliveira Paiva A.M., Kostidis S., Mayboroda O.A., Smits W.K., Krom B.P. (2016). Interspecies interactions between *Clostridium difficile* and *Candida albicans*. mSphere.

[17] MacAlpine J., Robbins N., Cowen L.E. (2023). Bacterial-fungal interactions and their impact on microbial pathogenesis. Mol. Ecol..

[18] Matsuda Y., Cho O., Sugita T., Ogishima D., Takeda S. (2018). Culture supernatants of *Lactobacillus gasseri* and *L. crispatus* inhibit Candida albicans biofilm formation and adhesion to Hela cells. Mycopathologia.

[19] Lee J.-H., Wood T.K., Lee J. (2015). Roles of indole as an interspecies and interkingdom signaling molecule. Trends Microbiol..

[20] Roager H.M., Licht T.R. (2018). Microbial tryptophan catabolites in health and disease. Nat. Commun..

[21] Di Martino P., Fursy R., Bret L., Sundararaju B., Phillips R.S. (2003). Indole can act as an extracellular signal to regulate biofilm formation of *Escherichia coli* and other indole-producing bacteria. Can. J. Microbiol..

[22] Wang Y., Tian T., Zhang J., Jin X., Yue H., Zhang X.-H., Du L., Bai F. (2019). Indole reverses intrinsic antibiotic resistance by activating a novel dual-function importer. mBio.

[23] Inaba T., Obana N., Habe H., Nomura N. (2020). Biofilm formation by *Streptococcus mutans* is enhanced by indole via the quorum sensing pathway. Microb. Environ..

[24] Liu Y.-Y., Chen H.-W., Chou J.-Y. (2016). Variation in indole-3-acetic acid production by wild *Saccharomyces cerevisiae* and *S. paradoxus* strains from diverse ecological sources and its effect on growth. PLoS ONE.

[25] Xing P.Y., Agrawal R., Jayaraman A., Martin K.A., Zhang G.W., Ngu E.L., Faylon L.E., Kjelleberg S., Rice S.A., Wang Y. (2024). Microbial indoles: Key regulators of organ growth and metabolic function. Microorganisms.

[26] Sun H., Zheng M., Song J., Huang S., Pan Y., Gong R., Lin Z. (2019). Multiple-species hormetic phenomena induced by indole: A case study on the toxicity of indole to bacteria, algae and human cells. Sci. Total Environ..

[27] Raut J.S., Shinde R.B., Karuppayil M.S. (2012). Indole, a bacterial signaling molecule, exhibits inhibitory activity against growth, dimorphism and biofilm formation in *Candida albicans*. Afr. J. Microbiol. Res..

[28] Zhang Y.-G., Zhang T., Lin L. (2024). Identification of flo11-like adhesin in *Schizosaccharomyces pombe* and the mechanism of small-molecule compounds mediating biofilm formation in yeasts. Microorganisms.

[29] Wolska K.I., Grudniak A.M., Rudnicka Z., Markowska K. (2016). Genetic control of bacterial biofilms. J. Appl. Genet..

[30] Graboski A.L., Kowalewski M.E., Simpson J.B., Cao X., Ha M., Zhang J., Walton W.G., Flaherty D.P., Redinbo M.R. (2023). Mechanism-based inhibition of gut microbial tryptophanases reduces serum indoxyl sulfate. Cell Chem. Biol..

[31] Do Q.T., Nguyen G.T., Celis V., Phillips R.S. (2014). Inhibition of *Escherichia coli* tryptophan indole-lyase by tryptophan homologues. Arch. Biochem. Biophys..

[32] Wuertz S., Hendrickx L., Kuehn M., Rodenacker K., Hausner M. (2001). In situ quantification of gene transfer in biofilms. Methods Enzymol..

[33] Odularu A.T., Afolayan A.J., Sadimenko A.P., Ajibade P.A., Mbese J.Z. (2022). Multidrug-resistant biofilm, quorum sensing, quorum quenching, and antibacterial activities of indole derivatives as potential eradication approaches. BioMed Res. Int..

[34] Jeong H., Boya B.R., Kim Y.G., Lee J.H., Lee J. (2025). Antifungal activities of multi-halogenated indoles against drug-resistant candida species. Int. J. Mol. Sci..

[35] Manoharan R.K., Lee J.H., Lee J. (2018). Efficacy of 7-benzyloxyindole and other halogenated indoles to inhibit *Candida albicans* biofilm and hyphal formation. Microb. Biotechnol..

[36] Ma J., Jiang Y., Zhuang X., Chen H., Shen Y., Mao Z., Rao G., Wang R. (2022). Discovery of novel indole and indoline derivatives against *Candida albicans* as potent antifungal agents. Bioorg. Med. Chem. Lett..

[37] Li G., Young K.D. (2013). Indole production by the tryptophanase *tnaA* in *Escherichia coli* is determined by the amount of exogenous tryptophan. Microbiology.

[38] Datsenko K.A., Wanner B.L. (2000). One-step inactivation of chromosomal genes in *Escherichia coli* k-12 using PCR products. Proc. Natl. Acad. Sci. USA.

[39] Meudt W.J., Gaines T.P. (1967). Studies on the oxidation of indole-3-acetic acid by peroxidase enzymes. i. colorimetric determination of indole-3-acetic acid oxidation products. Plant Physiol..

[40] O’Toole G.A. (2011). Microtiter dish biofilm formation assay. JoVE-J. Vis. Exp..

[41] Lee J.H., Kim Y.G., Lee J. (2024). Antibiofilm activity of lawsone against polymicrobial enterohemorrhagic Escherichia coli O157:h7 and *Candida albicans* by suppression of curli production and hyphal growth. Phytomedicine.

[42] van Genechten W., Van Dijck P., Demuyser L. (2021). Fluorescent toys ‘n’ tools lighting the way in fungal research. FEMS Microbiol. Rev..

[43] Lee J.H., Kim Y.G., Khadke S.K., Yamano A., Watanabe A., Lee J. (2019). Inhibition of biofilm formation by *Candida albicans* and polymicrobial microorganisms by nepodin via hyphal-growth suppression. ACS Infect. Dis..

[44] Breugelmans P., Barken K.B., Tolker-Nielsen T., Hofkens J., Dejonghe W., Springael D. (2008). Architecture and spatial organization in a triple-species bacterial biofilm synergistically degrading the phenylurea herbicide linuron. FEMS Microbiol. Ecol..

[45] Horemans B., Breugelmans P., Hofkens J., Springael D. (2017). Carbon catabolite repression and cell dispersal affect degradation of the xenobiotic compound 3,4-dichloroaniline in *Comamonas testosterone* WDL7 biofilms. FEMS Microbiol. Ecol..

[46] Pan Z., Dai C., Li W. (2024). Material-based treatment strategies against intraosseous implant biofilm infection. Biochem. Biophys. Rep..

