# Supplementary material for: Roles of Indole and Its Derivative in Modulating E. coli–Candida albicans Biofilm Formation
Source: Int J Mol Sci. 2026 May 16;27(10):4478. doi: 10.3390/ijms27104478 (PMC13208022; doi:10.3390/ijms27104478)
Supplement: Supplementary file 1 [file ijms-27-04478-s001.zip › ijms-4248878-supplementary.pdf]

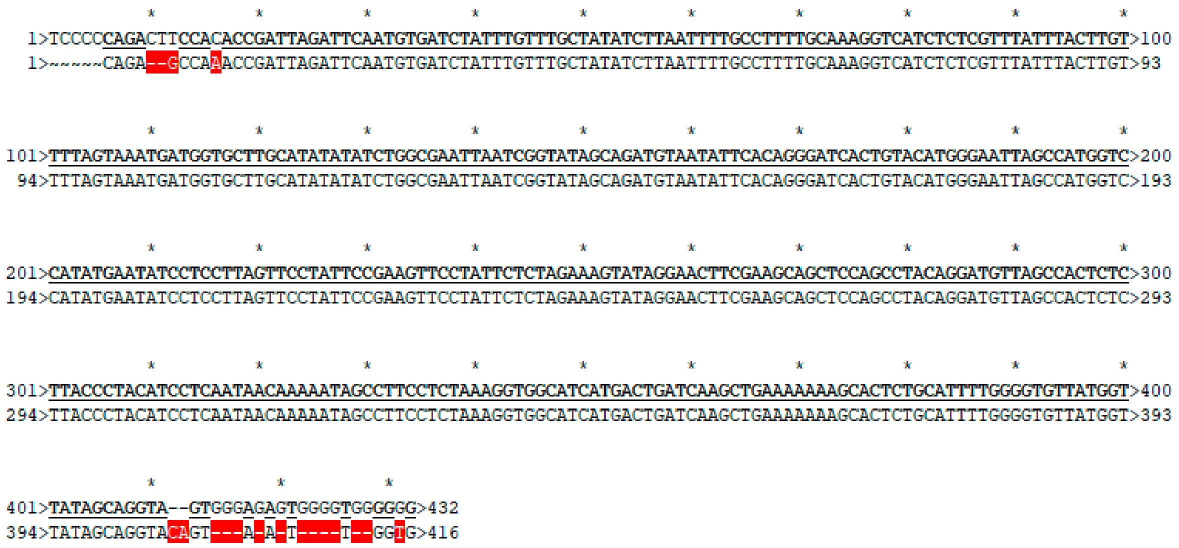

Figure S1 Sequencing verification of the *tnaA*Δ strain of *E. coli* BW25113 . The theoretical sequence is displayed above, and the experimental sequencing trace is shown below. Alignment confirms the successful construction of the *tnaA* knockout strain.

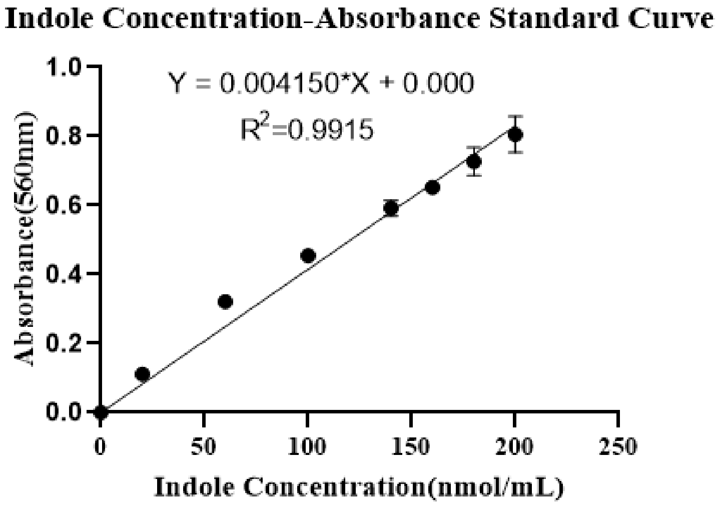

Figure S2 A standard curve generated by serially diluting the indole standard to different concentrations during the DMACA assay.

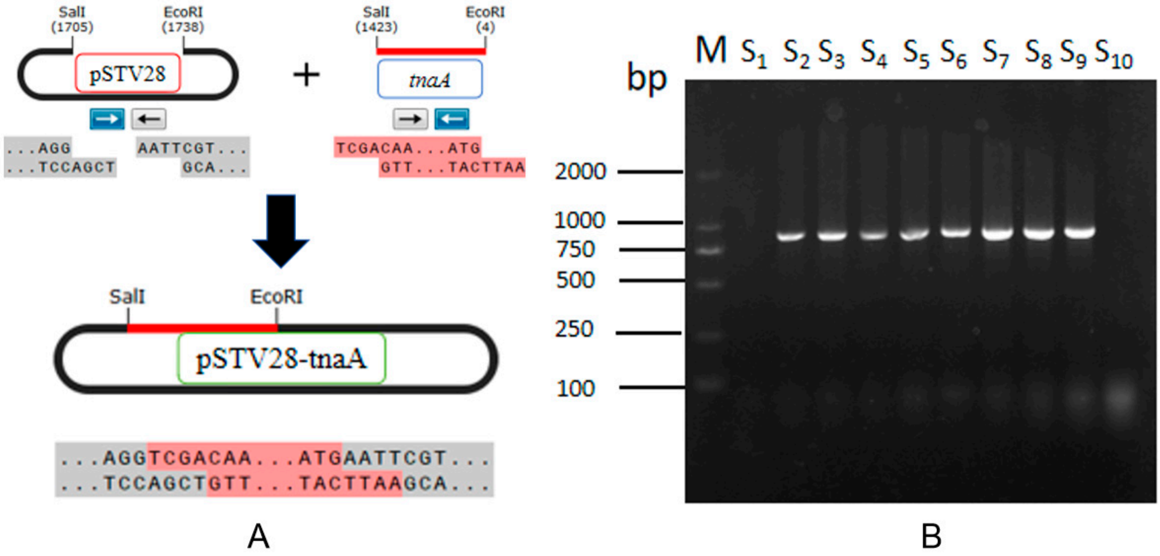

Figure S3 Construction of the *E. coli* complemented strain *tnaA*Δ/pSTV28-*tnaA*. (A) Schematic representation of pSTV28-*tnaA*; (B) Electrophoretic analysis of PCR verification for pSTV28-*tnaA*.

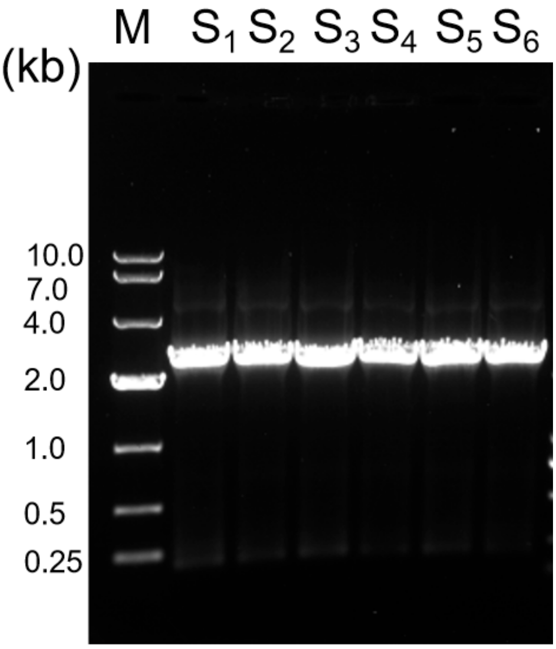

Figure S4. The *als3*-yeGFP homologous recombination fragment for *C. albicans* was PCR-amplified, and the band shown on gel electrophoretic image was 2.5 kb in size, as expected.

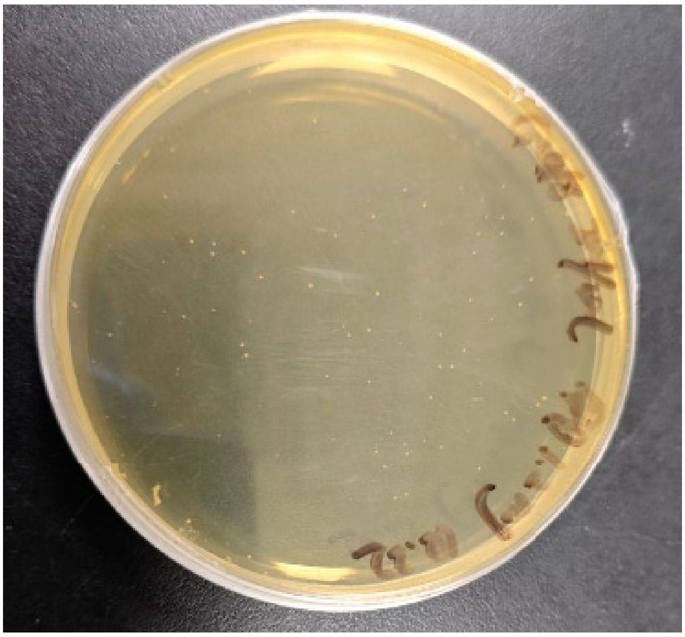

Figure S5. The *C. albicans als3*-yeGFP integration cassette was selected on G418-containing YPD agar with molybdate as the adjuvant for facilitating the screening of the transformants.

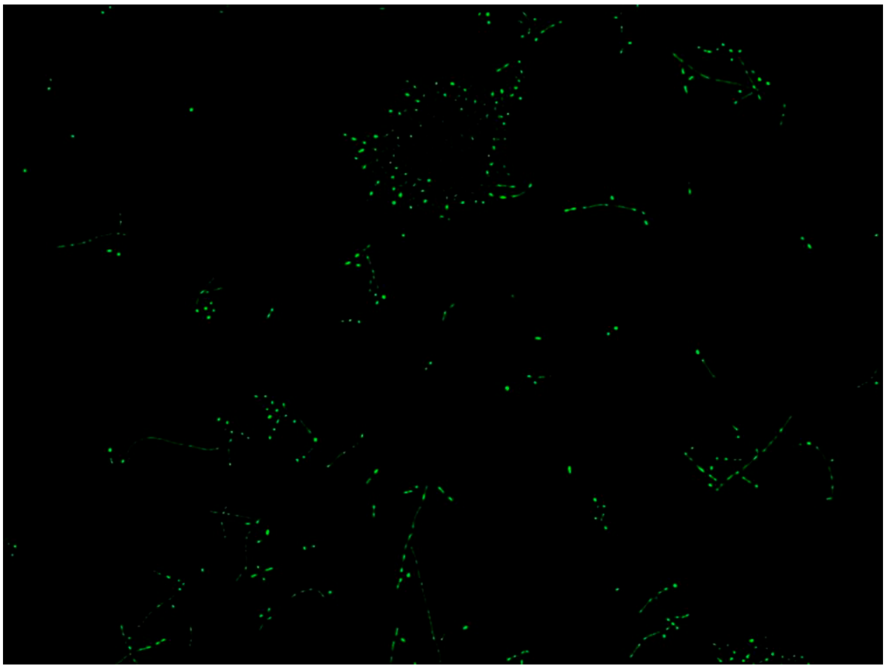

Figure S6 Preliminary examination of yeGFP fluorescence in *C. albicans*. Positive clones harboring the *als3*-yeGFP integration cassette were picked, cultured in a sterile 48-well microplate, and visualized using a live-cell imaging system.

Table S1. List of PCR primers used in the experimental procedures.

primer	sequence（5′-3′）
F1	ctggcgaattaatcggtatagcagatgtaatattcacagggatcactgtaCATGGGAATTAGCCATGGTCC
R1	aaggctatttttgttattgaggatgtagggtaagagagtggctaacatccTGTAGGCTGGAGCTGCTTCG
F2	CTCCTGTTATTCCTCAACCC
R2	ATATCCAGCTGAACGGTCTG
F3	CAGAGCCAAACCGATTAGAT
R3	CACCAATTACTGTACCTGCT
F4	CCGGAATTCATGGAAAACTTTAAACATCTCCCTGAACCG
R4	ACGCGTCGACAACTTCTTTAAGTTTTGCGGTGAAGTGACG
F5	CCTTAATTAAGATGTCTAAAGGTGAAGAATTATTCACTGGTG
R5	AGGCGCGCCTTTGTACAATTCATCCATACCATGGG
F6	tacatttgacggatctggttctgttattcaacattctacttggttatgtggtttgatcacattattatccttatttattCGGATCCCCGGGTTAATTAA
R6	taattttttttttggagccaaaaaaacaaaaaacaaacaaataacaaaaatctaaaaaggcgactatgatggtatcatccGAATTCGAGCTCGTTTAAAC

The purposes of all primers are listed below:

F1/R1: Amplification of the targeting fragment for *tnaA* knockout in *E. coli.*

F2/R2: Verification of the *tnaA* replacement with the resistance gene.

F3/R3: Verification of resistance cassette excision.

F4/R4: Amplification of *tnaA* for cloning into the complementation plasmid pSTV28.

F5/R5: Amplification of yeGFP for constructing the pFA6a-yeGFP-KanMX6 plasmid.

F6/R6: Generation of the homologous recombination fragment for *als3*-yeGFP integration in *C. albicans.*

Table S2. qPCR primer sequence.

Gene	Sequence (5′-3′)	PCR product size (bp)
hwp1	5′-TGGCTAGTGAAACCTCACC-3′<br>5′-GTTGCATGAGTGGAACTGATTC-3′	150
ece1	5′- CCAGAAATTGTTGCTCGTGTTG -3′<br>5′-CAGGACGCCATCAAAAACG -3′	138
als3	5′-GTGATGCTGGATCTAACGGTATTG-3′<br>5′- GTCTTAGTTTTGTCGCGGTTAGG -3′	112
act1	5′-TTGACCAAACCACTTTCAACTC-3′<br>5′-AGAAGATGGAGCCAAAGCAG-3′	152
